# Fitness Impact and Stability of a Transgene Conferring Resistance to Dengue-2 Virus following Introgression into a Genetically Diverse *Aedes aegypti* Strain

**DOI:** 10.1371/journal.pntd.0002833

**Published:** 2014-05-08

**Authors:** Alexander W. E. Franz, Irma Sanchez-Vargas, Robyn R. Raban, William C. Black, Anthony A. James, Ken E. Olson

**Affiliations:** 1 Department of Veterinary Pathobiology, University of Missouri, Columbia, Missouri, United States of America; 2 Arthropod-borne and Infectious Diseases Laboratory, Department of Microbiology, Immunology and Pathology, Colorado State University, Fort Collins, Colorado, United States of America; 3 Departments of Microbiology and Molecular Genetics and Molecular Biology and Biochemistry, University of California, Irvine, California, United States of America; University of Perugia, Italy

## Abstract

In 2006, we reported a *mariner* (*Mos1*)-transformed *Aedes aegypti* line, Carb77, which was highly resistant to dengue-2 virus (DENV2). Carb77 mosquitoes expressed a DENV2-specific inverted-repeat (IR) RNA in midgut epithelial cells after ingesting an infectious bloodmeal. The IR-RNA formed double-stranded DENV2-derived RNA, initiating an intracellular antiviral RNA interference (RNAi) response. However, Carb77 mosquitoes stopped expressing the IR-RNA after 17 generations in culture and lost their DENV2-refractory phenotype. In the current study, we generated new transgenic lines having the identical transgene as Carb77. One of these lines, Carb109M, has been genetically stable and refractory to DENV2 for >33 generations. Southern blot analysis identified two transgene integration sites in Carb109M. Northern blot analysis detected abundant, transient expression of the IR-RNA 24 h after a bloodmeal. Carb109M mosquitoes were refractory to different DENV2 genotypes but not to other DENV serotypes. To further test fitness and stability, we introgressed the Carb109M transgene into a genetically diverse laboratory strain (GDLS) by backcrossing for five generations and selecting individuals expressing the transgene's EGFP marker in each generation. Comparison of transgene stability in replicate backcross 5 (BC_5_) lines versus BC_1_ control lines demonstrated that backcrossing dramatically increased transgene stability. We subjected six BC_5_ lines to five generations of selection based on EGFP marker expression to increase the frequency of the transgene prior to final family selection. Comparison of the observed transgene frequencies in the six replicate lines relative to expectations from Fisher's selection model demonstrated lingering fitness costs associated with either the transgene or linked deleterious genes. Although minimal fitness loss (relative to GDLS) was manifest in the final family selection stage, we were able to select homozygotes for the transgene in one family, Carb109M/GDLS.BC_5_.HZ. This family has been genetically stable and DENV2 refractory for multiple generations. Carb109M/GDLS.BC_5_.HZ represents an important line for testing proof-of-principle vector population replacement.

## Author Summary

Expression of a DENV2 sequence-derived IR RNA in the mosquito midgut initiates an antiviral intracellular RNAi response that efficiently blocks DENV2 infection and profoundly impairs vector competence for that virus in *Aedes aegypti*. DENV2-specific IR RNA expression in the Carb109M strain has maintained the RNAi-based, refractory phenotype for 33 generations in laboratory culture. The two transgene integration sites were stable after multiple generations and following introgression into a genetically-diverse (GDLS) *Ae. aegypti* population. Introgression of the transgene into the GDLS genetic background changed GDLS from a DENV2 susceptible phenotype to a DENV2 refractory phenotype. The DENV2 refractory homozygous line, Carb109M/GDLS.BC_5_.HZ, exhibits (relative to GDLS) minimal fitness loss associated with the transgene. This line could be a potential candidate for proof-of-principle field studies.

## Introduction

The four serotypes of dengue viruses (DENV1-4; *Flaviviridae; Flavivirus*) are considered the most important mosquito-transmitted arboviruses infecting humans. Epidemiologists have estimated 100–390 million people per year acquire DENV infections in tropical and subtropical regions of the world [1], [2]. Dengue disease symptoms range from mild febrile illness, referred to as dengue fever (DF), to severe disease dengue hemorrhagic fever (DHF) and dengue shock syndrome (DSS) [3]. DENV prevalence is increasing rapidly throughout South-East Asia, and Central-and South-America due to rapid urbanization, increased trade and human traffic. DENV in these regions can be hyper-endemic [4], further increasing the risk of DHF. Furthermore, virulent strains have been introduced in the last decades from South-East Asia into Central-America and the Caribbean replacing endogenous DENV2 genotypes and causing more cases of DHF and DSS among local populations [5]–[7]. Currently, there are no vaccines or therapeutic drugs readily available to the more than two billion people at risk for DENV infection or the tens of millions manifesting some level of disease [2], [8]. Thus, DENV prevention relies on vector control through indoor insecticide spraying, using insecticide treated door/window curtains and reducing the number of potential oviposition sites [9]–[11].

The principal vector of DENV is the peridomestic mosquito, *Aedes aegypti* (L.), which is distributed widely in many regions of the world and is a major factor contributing to the global incidence of DEN disease. Novel alternative vector control strategies are now being tested that use genetically-modified *Ae. aegypti* carrying a dominant-lethal gene (RIDL) to reduce mosquito populations [12]–[14]. A second novel concept in DEN disease control is replacement of DENV-competent mosquito populations with DENV-refractory vectors [15]–[17]. The work presented here describes the generation of a new *Ae. aegypti* transgenic strain, Carb109M/GDLS.BC_5_.HZ, which expresses an anti-DENV2 gene construct and is highly refractory to the virus after being introgressed into a genetically diverse laboratory strain (GDLS).

*Ae. aegypti* females acquire a DENV-containing bloodmeal from a viremic human host. DENV initially infects midgut epithelial cells and 4–5 days later disseminates to hemocytes, fat body, nervous system tissues, and salivary glands. The mosquito can transmit virus to a new host 10 to 14 days post-infection (dpi) depending on ambient conditions, virus strain and mosquito competence [18], [19]. DENV is confronted inside the mosquito cell by the innate antiviral, exogenous small interfering RNA (siRNA) pathway [20]. The antiviral, siRNA arm of the RNAi pathway is a major defense used by mosquitoes early in infection with arboviruses [21]. The sequence-dependent RNAi pathway has been described in great detail [22]. During a typical mosquito infection with DENV, 21 nt virus-derived siRNAs (or viRNAs) are readily detectable, indicating that the RNAi machinery degrades viral genomes [23]–[25]. We showed that DENV titers increase in vectors when the RNAi pathway is impaired leading to significantly higher midgut infections and dissemination rates and shorter extrinsic incubation periods [23], [26], [27]. Thus, the RNAi pathway modulates DENV replication in the mosquito and may keep virus concentrations below a threshold that could become detrimental to insect fitness.

Even though *Ae. aegypti* has a highly functional antiviral RNAi pathway, the vector remains an efficient transmitter of DENV. DENVs may have evolved mechanisms to counter the mosquito antiviral RNAi response. Schnettler and colleagues reported that the 3′UTR of the DENV genome generates a subgenomic flavivirus RNA (sfRNA) that modulates RNAi as part of a counter-defense [28]. Our strategy has been to initiate a DENV2-specific RNAi response in midgut epithelial cells within the first few hours following acquisition of a viremic bloodmeal and thereby prevent the virus from establishing infection foci in these cells. This RNAi-mediated midgut infection barrier should prevent further accumulation of sfRNAs or other RNAi-modulating factors the virus uses to counteract the RNAi response. We hypothesize that perturbing homeostasis between vector RNAi defense and virus counter-response and establishing a specific antiviral RNAi response coincident with infection will reduce vector competence and lower disease transmission.

The successful use of genetically-modified mosquitoes for gene replacement in the field requires stable effector gene expression over many generations and across diverse genetic backgrounds to maintain the DENV refractory phenotype. Furthermore, effector genes need to be designed that target each of the four DENV serotypes and the many different DENV genotypes that arise among serotypes. Ideally, these transgenes should have minimal fitness costs relative to wild-type mosquitoes or the transgenic mosquito is unlikely to spread the effector gene through the population [29]–[31]. Earlier studies suggest evidence of a fitness load associated with transgenes [32]–[34]. However, these studies failed to assess this effect in different genetic backgrounds. Without this information the effect of the transgene cannot be distinguished from the generally low fitness imposed by deleterious recessive alleles as they become homozygous in inbred lines. Assessing transgene loads absolutely requires its analysis in diverse genetic backgrounds [30].

Previously, we described the generation of a transgenic *Ae. aegypti* line, Carb77, which was engineered to be refractory to DENV2 by expressing an IR effector RNA in midgut tissue for a 24–48 h period after receiving a bloodmeal [16]. Carb77 mosquitoes exhibited a strong midgut infection barrier for DENV2. However, Carb77 mosquitoes eventually lost their refractory phenotype for DENV2 [17]. The transgene sequence was fully intact in Carb77 G_17_, but the IR RNA was no longer detected by Northern blot analysis [17]. We speculated that the loss of IR-RNA expression could have been due to chromatin/heterochromatin rearrangements that enabled regulatory elements of neighboring or even distant genes to silence the transgene [35].

In this study we engineered new transgenic *Ae. aegypti* lines harboring the same transgene as line Carb77 to further evaluate genetic and phenotypic stability associated with the transgene. One of these lines, Carb109M, was highly refractory to DENV2 infection. To evaluate the effect of genetic background on transgene stability, we performed five generations of backcrossing into a genetically-diverse laboratory strain (GDLS) derived from field collections of *Ae. aegypti* from southern Mexico [13], [36]. We selected for the EGFP eye marker associated with the transgene in each of the five backcross generations to produce six BC_5_ lines. The BC_5_ lines were subject to five generations of selection for the EGFP eye marker to increase the frequency of the transgene prior to final family selection. We were able to select one family, Carb109M/GDLS.BC_5_.HZ,that has maintained the DENV2 refractory phenotype for multiple intercrossed generations. Our results illustrate clearly the importance of outcrossing transgenes into genetically diverse, preferably recently-colonized, strains before attempting to assess transgene-associated fitness loads and certainly before using genetically modified strains in population cage or field experiments.

## Methods

### Mosquito rearing and maintenance

All *Ae. aegypti* colonies were maintained in a BSL2/Arthropod Containment Level 2 (ACL2) insectary as described [16]. The temperature in the insectary was 28°C with 75–80% relative humidity and a 12 h light/12 h darkness cycle. Adults were generally maintained in 1ft^3^ cages and fed on raisins. For routine rearing and maintenance, mated females (approximately 1-week post-eclosure) received artificial bloodmeals consisting of citrated sheep blood. Females were allowed to oviposit eggs on paper towel strips inserted into small water-filled oviposition cups. The eggs deposited on papers (or eggliners) were viable for up to 3–4 months before hatching. Eggs were hatched in pans containing sterile, de-aerated water to synchronize the hatch and larvae were fed ground TetraMin (Melle, Germany) aquarium food. Pupae were separated and placed into water-filled cups. Pupae identified as females based upon size were mixed with male pupae at a ratio of ∼1∶20. Two cups with up to 500 female pupae were placed in a 1ft^3^ cage prior to eclosion.

### Transgene construction and generation of transgenic *Ae. aegypti*

**Figure 1:**
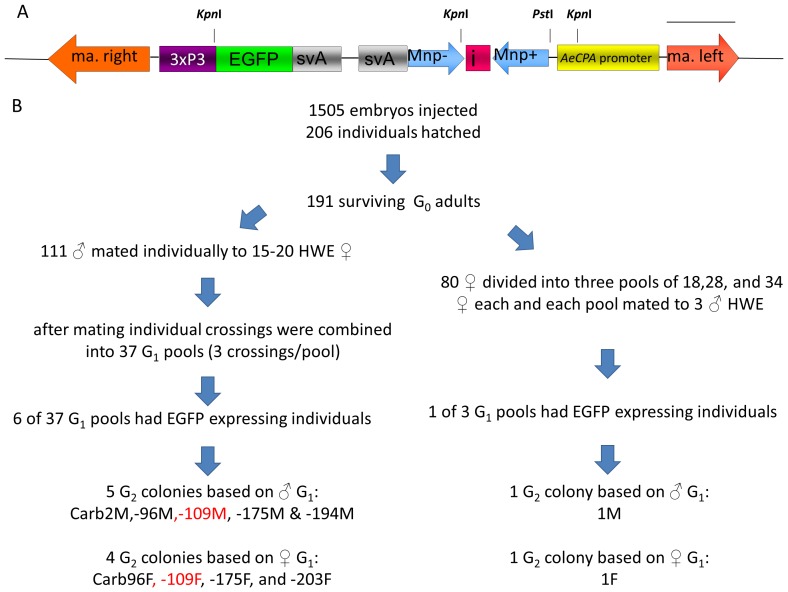
Generation of transgenic *Ae. aegypti* expressing an IR-RNA targeting DENV2 in midguts of bloodfed females. (A) Schematic representation of the transgene based on the *mariner Mos1* TE [16], [17]. The endonuclease restriction sites used for the Southern blot analysis are indicated. The cDNA probe of the Southern blot corresponded to the left arm of *Mos1*. Abbreviations: ma.right or ma.left  =  *mariner* (*Mos1*) right or left arm; 3xP3 =  eye-specific synthetic promoter; svA  =  SV40 poly A signal; Mnp  = M (no protein) and includes DENV2 prM-M coding sequence preceded with stop codons to prevent translation; Mnp (−) or (+)  =  orientation of sequence to transcribe IR-RNA (with respect to the *AeCPA* promoter); i =  intron sequence; *AeCPA*  =  *Ae. aegypti carboxypeptidase A* promoter; EGFP  =  enhanced green fluorescent protein. (B) Flowchart showing the establishment of transgenic mosquito lines based on pooling of individual families containing transgenic founders. HWE  =  Higgs white-eye *Ae. aegypti* line.

The *mariner Mos1*-based IR effector gene construct (*p*Mos-carb/Mnp/i/Mnp/svA) was used to transform pre-blastoderm embryos of the Higgs White Eye (HWE) strain of *Ae. aegypti* [37]. The *Mos1*-based DNA constructs were identical to those used to generate Carb77 mosquitoes earlier (Fig. 1A) [16]. The inverted-repeat effector RNA (IR-RNA) sequence was derived from nucleotide position nt 401–969 (prM-M/E coding region of DENV2-Jamaica1409 RNA genome sequence; GenBank: M20558.1). Microinjection of donor (*p*Mos-carb/Mnp/i/Mnp/svA) and *mariner Mos1* transposase helper plasmids has been described [16], [38]. Methods used to screen for transformation and identify new transgenic lines were identical to those described previously [16], [38]. Surviving G_0_ adults were outcrossed to the HWE recipient strain and pooled to reduce the overall number of subsequent bloodfeeds as described in Fig. 1B and Table 1. Eleven separate colony lines containing transgenic G_1_ individuals were formed and designated Carb1M, Carb1F, Carb22M, Carb96M, Carb96F, Carb109M, Carb109F, Carb175M, Carb175F, Carb194M, and Carb203F (Table 1). Following generation G_4_, mosquito lines were increased through sib-matings. In the Method descriptions, we generically refer to the HWE-based transgenic lines as HTLs. The Carb52 transgenic line has no anti-DENV effector gene but does express EGFP in the midgut after a bloodmeal [39]. Carb52 was used as a DENV2-susceptible, transgenic control in virus prevalence assays.

**Table 1:** Overview of transgenic *Ae. aegypti* (pools and lines) generated containing the *p*Mos- carb/Mnp/i/Mnp/svA transgene.

Pooled G_0_ males	G_0_ pool designation	Number/sex of transgenic G_1_ after outcrossing to HWE	Transgenic lines maintained as separate colonies (from G_1_ onwards)	Carb109M and Carb109F after one, five, or six backcrosses into GDLS genetic background
20-21-22	P22	single male	Carb22M†	
96-97-98	P96	multiple males, females	Carb96M*, Carb96F†	
109-110-111	P109	multiple males, females	Carb109M, Carb109F	Carb109M/GDLS.BC_1_
				Carb109F/GDLS.BC_1_
				Carb109M/GDLS.BC_5_
				Carb109F/GDLS.BC_5_
				Carb109M/GDLS.BC_6_
				Carb109F/GDLS.BC_6_
175-177-179	P175	multiple males, females	Carb175M*, Carb175F**	
194-195-196	P194	single male	Carb194M††	
203-204-206	P203	single female	Carb203F††	
**Pooled G_0_ females**				
1 (34 females)	P1	multiple males, females	Carb1M†, Carb1F†	

*Transgenic lines were lost in a subsequent next generation prior to analysis of DENV2 refractory phenotype.

† Transgenic lines discontinued due to lack of a DENV2 refractory phenotype.

**Transgenic line discontinued after initial characterizations. Line initially had DENV2 refractory phenotype, but phenotype declined in subsequent generations.

†† Transgenic lines discontinued; lines initially had DENV2 refractory phenotype, but resistance was lost in subsequent generations.

### Northern blot analysis

Effector gene expression in HTLs was characterized by Northern blot analysis. Total RNA was extracted using TRIzol (Invitrogen, Carlsbad, CA) from a pool of 20 midguts originating from female mosquitoes given a non-infectious bloodmeal. RNA was analyzed at 20 and 48 h post-bloodmeal or from mosquitoes receiving only sugar. Approximately 3–5 µg of pooled midgut RNA was separated on a 1.2% agarose gel by electrophoresis and blotted onto a positively-charged nylon membrane (Applied Biosystems). Blots were hybridized overnight at 52°C with a random-primed ^32^P-dCTP labeled DNA probe (Megaprime DNA Labelling Kit, Amersham Biosciences, NJ), corresponding to the prM-M encoding region of the DENV2-Jamaica1409 RNA genome.

### Southern blot analysis

Effector gene integration in HTL mosquitoes was characterized initially by Southern blot analysis [40]. Each sample of total DNA was extracted from three females using the Puregene kit (Qiagen, Valencia, CA). DNA pellets were suspended in 50 µl Hydration Solution overnight at room temperature. Approximately 5 µg of total DNA were digested with restriction endonucleases *Kpn*I or *Pst*I for 4 h at 37°C. DNAs were denatured and separated by electrophoresis on a 0.8% agarose gel and transferred to a positively-charged nylon membrane (‘Brightstar’, Applied Biosystems, CA). Blots were hybridized with a [α-^32^P]dCTP- (3,000 Ci/mmol) labeled probe corresponding to the 354 bp left arm of *mariner Mos1* DNA generated with the DECAprime II Random Primed Labeling kit (Applied Biosystems). Hybridizations were performed overnight at 48°C.

### Genome walking procedure

The integration site of the transgene in a selected HTLs was determined using the Clontech GenomeWalker Universal kit (Takara Bio Company, Mountain View, CA) and Advantage2 polymerase mix [17], [26], [39]. Total genomic DNA was extracted from transgenic males and females using the DNeasy Blood & Tissue Kit (Qiagen, Valencia, CA). DNA was digested with *Dra* I, *Eco*R V, *Pvu* II, or *Stu* I, and ligated to the GenomeWalker adaptor provided with the kit. Amplification products were generated using the Advantage2 polymerase (Takara Bio Company). An initial amplification reaction was conducted using the outer *mariner Mos1*-specific primers maLeft FWD: 5′caattatgacgctcaattcgcgccaaac3′, maRight REV: 5′gagcagcgcttcgattcttacgaaagtgtg3′, and the forward and reverse adaptor primers of the Genome Walker kit [17]. The resulting amplicons were used to generate nested PCR amplifications. The *mariner Mos1*-specific primers for the nested PCR reactions were: maRight_nested REV 5′gacgatgagttctactggcgtggaatcc3′ and maLeft_nested FWD 5′gtggttcgacagtcaaggttgacacttc3′and the forward- and reverse-nested adaptor primers of the Genome Walker kit. Amplicons were inserted into the TOPO-TA cloning vector (Invitrogen, Carlsberg, CA) and sequenced using TOPO-TA vector-specific primers. The transgene integration and orientation within the AAEL010318 gene (VectorBase supercontig 1.470) were confirmed in a standard PCR reaction using 500 ng of total DNA of a HTL mosquito and primer pairs 10318 FWD (5′ctcacacggcattacatgaaatatgttagtatttaatc3′)/maRight REV and 10318 REV (5′aacagtagcttgtatgcttaggcatactaattgag3′)/maLeft FWD.

### siRNA profiling

Females from a selected HTL were offered a non-infectious bloodmeal. Total RNA was extracted 20 h post-bloodmeal from pools of 50 midguts using TRIzol reagent (Invitrogen). Total RNA was size-fractionated using the FlashPAGE fractionator (Applied Biosystems). Small RNA libraries were made using the SOLiD small RNA expression kit (Applied Biosystems) and sequenced on a SOLiD 3 sequencer (Applied Biosystems). Sequence data were analyzed using the NextGENe software package (Softgenetics, LLC, State College, PA), Version 1.96. Potential siRNAs were aligned to the DENV2 genome using the transcriptome assembly function. The reference genome was a FASTA file of DENV2-Jamaica1409 RNA.

### Arbovirus challenge experiments

DENV isolates used in this study were: Jamaica1409, C-932/Acapulco 97 (AY449678), Mex96 Merida (AY449677.1), QR94 Quintana Roo (AY449676; JX966379), and 14757 Yucatan [7], [19], [41] representing Asian 2, Cosmopolitan, American, and American-Asian genotypes of DENV2, respectively, and the 6889/QR-MX/97 isolate of DENV3 (DQ341205). DENVs were propagated in C6/36 cells at 0.01 multiplicity of infection (m.o.i.) for 12 days using Dulbecco's Modified Eagle Medium (DMEM) complemented with 3% fetal bovine serum (FBS). The cell culture medium was replaced after a 6 day incubation period at 28°C. Virus in cell culture media was collected at 12 dpi and mixed with defibrinated sheep blood at a 1∶1 ratio. Females were fed for 1 h with the cell culture-blood mixture at 37°C using a single glass feeder per carton [16], [23]. Engorged females were selected after bloodfeeding, reared in 1.9 L (64 oz.) cartons and offered sucrose and water until further analysis. Chikungunya virus (CHIKV; *Alphavirus*) 37997 (GenBank accession: AY726732.1) was propagated in Vero cells for 36 h at an m.o.i. of 0.01 using Minimum Essential Medium Eagle (MEM) complemented with 7% FBS. Bloodfeeding with virus was performed as described above for mosquito infections with DENVs [16].

### Arbovirus detection

Virus titers from individual mosquitoes were determined by plaque assay at 7 and 14 dpi [16]. Samples were homogenized in 0.5 ml 7% FBS-complemented DMEM and MEM medium, respectively. Homogenized samples were filtered with Acrodisc HT Tuffryn 0.2 µm syringe filters (Pall Life Sciences, East Hills, NY). Vero cells (for CHIKV) or LLC-MK2 cells (for DENV) were seeded into 24-well plates and left for three days to achieve confluence. Cells were infected with 10-fold serial dilutions of each mosquito homogenate. Cells were incubated for 1 h at 37°C before overlaying with an agarose nutrient mixture [1x Medium 199 (Sigma-Aldrich, St. Louis, MO), 10% FBS, 4% NaHCO_3_, 0.5% MEM vitamins, 0.5% MEM amino acids (Mediatech Inc., Manassas, VA); 1% Hanks-DMEM medium (only used for DENV)]. Plates were incubated at 37°C for 4 and 12 days for CHIKV and DENV, respectively. Cells then were stained with MTT (3-[4,5-dimethylthiazol-2-yl]-2,5-diphenyltetrazolium bromide) (Sigma-Aldrich, St. Louis, MO), incubated at 37°C for 24 h and the number of plaques counted for each sample. Viral titers of individual mosquitoes were calculated as plaque forming units per milliliter (pfu/mL).

### Introgression of the transgene from selected HTLs into a diverse genetic background

GDLS mosquitoes were bred by mixing equal numbers of larvae from 10 separately-maintained *Ae. aegypti* populations from Chiapas State, Mexico [13]. Five larvae from each of the 10 strains were placed into 1 liter of tap water, poured into a 4 liter plastic box and fed as described above. The resulting adult females were mated to males from the same box and then bloodfed. The eggs arising from these matings were used in the initial HTL x GDLS intercrosses and in each of the subsequent five backcross generations. Virgin HTL females were placed in a cage with virgin GDLS males in each backcross, and reciprocally, virgin HTL males were placed in a separate cage with virgin GDLS females. Females were bloodfed and allowed to oviposit. Eggs were hatched, raised to third instar larvae and larvae lacking EGFP eye marker expression (wild-type homozygotes) were counted and culled. Two hundred larvae expressing EGFP were reared to adults and virgin adults then were mated to virgin GDLS. This backcross procedure was repeated four more times to yield HTL/GDLS.BC_5_. Theoretically, five generations of backcrossing should generate mosquitoes in which 31 of every 32 alleles (97%) are expected to have originated from the GDLS strain. However, this is only true for those alleles unlinked to the HTL transgene [42]. Proportions of EGFP-expressing larvae in both HTL/GDLS.BC_1_ through BC_5_ were computed and compared by estimating the 95% Highest Density Interval (HDI) with WinBUGS [43] and the Credible Intervals for Proportions script [44]. An additional backcross (BC_6_) was generated for some analyzes described in the Results section.

### Transgene frequencies in HTL (BC_1_ and BC_5_)

Due to the number of backcrosses and the selection scheme in this study we limited our analysis of transgene frequency to two HTL lines generically termed here as HTL1 and HTL2. Selection of HTL1 and HTL2 was based on the two lines having the strongest, most consistent DENV2 refractory phenotype. After the first backcross, four groups of fifteen lines were initiated to assess whether backcrossing affected transgene stability. The first set of 15 lines was initiated by crossing a HTL1/GDLS.BC_1_ heterozygote with a GDLS parent to generate offspring having an initial HTL1allele frequency of 0.25. The offspring were allowed to inter-mate randomly. The expected frequency of EGFP-positive offspring through five generations (F_1_–F_5_) was 0.4375, based on Hardy-Weinberg expectations (0.25^2^ transgene homozygotes +2*0.25*(1–0.25) transgene heterozygotes). The second set of 15 lines was generated by intercrossing HTL1/GDLS.BC_1_ heterozygotes so that the initial frequency of the HTL1 allele was 0.5. The expected frequency of EGFP-expressing larvae in F_1_–F_5_ was 0.75 (0.5^2^ transgene homozygotes +2*0.5*(1–0.5) transgene heterozygotes). The third and fourth sets of 15 lines were the same as for the first and second sets except that HTL2/GDLS.BC_1_ were used. These 60 lines were maintained without selection of EGFP expressing larvae for five generations and the frequency of EGFP larvae was estimated in ∼150 larvae from each of the 15 lines in each generation. This same process was repeated for the BC_5_ offspring.

The relative fitness loads of the transgene in homozygotes, heterozygotes and fitness of wild-type homozygotes were estimated by identifying the fitness coefficients in Fisher's Selection Model [45] that most closely fit the observed proportion of larvae expressing EGFP (pEGFP_t_) in each of the six generations. Fisher's Model is:
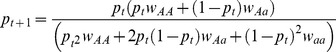
(1)where: p_t_ =  transgene frequency in generation t, w_AA_ =  fitness of transgene homozygotes, w_Aa_ =  fitness of transgene heterozygotes and w_aa_ =  fitness of wild-type homozygotes. A FORTRAN program was written that generated a three-dimensional matrix containing all combinations of W_AA_, W_Aa_ and W_aa_ each incremented by 0.01 from 0.0–1.0. The matrix therefore contained 100×100×100 = 10^6^ combinations. Fisher's model was run for five generations starting with a p_0_ (starting allele frequency) of either 0.5 or 0.25 for each combination. p_t_ were transformed into proportion predicted EGFP expressing larvae in each generation *t* (pEGFP_t_) by:



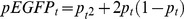
(2)All six values of pEGFP_t_ were compared with observed proportions oEGFP_t_ for t = 0….5 generations as:



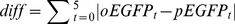
(3)The FORTRAN program identified the smallest *diff* and reported the associated w_AA_, w_Aa_ and w_aa_ values. Statistical comparisons among groups, generations and backcrosses were based on calculating Bayesian 95% Highest Density Intervals (95% HDI) using WinBUGS [43] and the estimation of mean and variance script [44]. Proportions with non-overlapping 95% HDI were considered credibly different.

### Relative fitness of HTL1/GDLS.BC_5_ and HTL2/GDLS.BC_5_ mosquitoes

Three crosses between HTL1/GDLS.BC_5_ heterozygotes (200 individuals/cross; F.1, F.2, F.3) and three crosses between HTL2/GDLS.BC_5_ heterozygotes (200 individuals/cross; M.1, M.2, M.3) were performed in six separate 1ft^3^ cages. One-week-old females received non-infectious bloodmeals as described above. All larvae from each cross were screened for EGFP expression and all wild-type larvae were culled. EGFP-expressing F_1_ larvae were reared to adults. Following random mating, females received bloodmeals and their F_2_ progeny were again screened for EGFP expression and wild-type larvae were culled. This procedure was followed for three more generations (F_3_–F_5_) to increase the frequency of the HTL1 or HTL2 transgene in the population while minimizing inbreeding. Results were again compared to values expected under Fisher's Model (eq. 1). Only w_AA_ and w_Aa_ were estimated because w_aa_  = 0 since all wild-type larvae were discarded during the selection process. Frequencies of EGFP-expressing larvae were recorded for five generations for each of the six lines. Mean observed-to-expected proportions were compared by estimating the 95% HDI with WinBUGS and the Estimating proportions script [44].

### Family-based selection to generate homozygous line based on HTL/GDLS.BC_5_

At the end of five generations of selection and assuming W_AA_  = W_Aa_  = 1, Fisher's model simplifies to
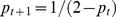
(4)and predicts that 98.22% of larvae were expected to express EGFP, and the transgene frequency was expected to be 0.83. Another 25 generations of selection would be required before nearly all larvae (99.9%) could be expected to express EGFP. We therefore switched to a family based selection scheme.

To generate HTL homozygous (HZ) families from the six lines (HTL1/GDLS.BC_5_ F.1, F.2, F.3 and HTL2/GDLS.BC_5_ M.1, M.2, M.3), 30 families were established each consisting of three F_5_ females placed in a cage with one male. Siblings from each of the thirty families were screened for EGFP expression and families with all siblings expressing EGFP were reared to adults and intercrossed. These offspring were reared to adults, intercrossed, bloodfed, eggs collected and hatched. These offspring were again screened for EGFP expression. Families, in which all siblings expressed EGFP were combined and maintained as homozygous (HZ) lines for further experiments. Ultimately this process yields one or more HTL/GDLS.BC_5_.HZ lines which can be tested for vector competence to DENV2-Jamaica1409.

## Results

### Generation of transgenic *Ae. aegypti* expressing an IR RNA to target DENV2 in midgut tissue

Transgenic lines were generated using the transgene described previously [16] to test whether the RNAi-based DENV2 refractory phenotype in Carb77 could be repeated and this time be genetically- and phenotypically-stable over time. We co-injected 1,505 pre-blastoderm HWE embryos with the *p*Mos-carb/Mnp/i/Mnp/svA donor and *mariner Mos1* transposase helper plasmids (Fig. 1A, B). We obtained 206 G_0_ larvae of which 191 developed into fertile adults. Each of the 111 G_0_ males was mated with 15–20 HWE females. Following a 2–3 day mating period, three of the crossings were combined into one pool each, reducing the number of 111 single crossings to 37 pools (Fig. 1B). The 80 G_0_ females were combined into three pools, each containing three HWE males.

Three eggliners were generated from each of the 40 pools. Larvae expressing the EGFP marker were observed among seven pools: P1, P22, P96, P109, P175, P194, and P203 (Table 1). All pools except P1 were based on male transgenic G_0_ founders (originating from micro-injected embryos). Transgenic male (M) and/or female (F) G_1_ mosquitoes from each pool were outcrossed separately to HWE resulting in 11 lines (Table 1; column 4) and their progeny were reared as separate colonies. Lines Carb96M and Carb175M were lost in a subsequent generation and before we could analyze their DENV2 refractory phenotypes (Table 1).

### DENV2 challenges of transgenic lines

The remaining nine transgenic lines were initially screened to identify which of them had a refractory phenotype for DENV2 infection. Mosquitoes from each line were offered bloodmeals containing 1.5×10^6^–1.6×10^7^ pfu/mL of DENV2-Jamaica1409. Fully-engorged transgenic G_3_ females were analyzed for DENV2 at 7 and 14 dpi by performing plaque assays in LLC-MK2 cells (Fig. S1A, B). DENV2 prevalence in Carb1M, Carb1F, Carb22M, and Carb96F at 7 and 14 dpi was not significantly different from that observed in the DENV2 competent control lines (HWE and Carb52) and as a consequence these lines were not further used in this study (Table 1). DENV2 prevalence in lines Carb194M and Carb203F was significantly lower than prevalence in HWE and Carb52 controls (Carb194M, p<0.01; Carb203F, p<0.02), but these lines were lost in subsequent generations (Table 1). In contrast, transgenic lines Carb109M, Carb109F, and Carb175F were highly resistant to DENV2 infection and exhibited a significantly lower DENV2 prevalence than control mosquitoes (Carb175F, p<0.004; Carb109M and Carb109F: p<0.0001). Further, all three lines were readily maintained in colony. However, in later generations Carb175F mosquitoes displayed a significantly weaker DENV2 refractory phenotype than Carb109M and Carb109F (data not shown; Table 1). Consequently, Carb175F was not used in the introgression studies. Carb109F and Carb109M were maintained as separate lines throughout this study because the two lines arose from a G_1_ female (F) and male (M) of pool 109, respectively. After further characterizations of the two lines having strong DENV2 refractory phenotypes, we selected Carb109F and Carb109M as HTL1 and HTL2 described in Methods for introgression and fitness analysis.

### IR-RNA expression in Carb109M and Carb109F mosquitoes

Northern blot analysis detected a single hybridizing moiety in Carb109M and Carb109F total midgut RNA 20 h after mosquitoes had received a non-infectious bloodmeal. This corresponded to the expected 568 nt size of the mature IR-RNA (Fig. S2A). The signal was not detected in RNA samples extracted at 48 h post- non-infectious bloodmeal or in RNA samples obtained from sugar-fed females. IR RNA also was not detected in the midguts of HWE or Carb52 lines [39]. Similarly to Carb109M and Carb109F, we detected IR RNA expression by northern blot analysis in Carb175F midguts at 20 h post bloodmeal (Fig. S2B). However, the hybridization signal was faint even after long exposure times of the northern blot and consistent with the weaker virus resistant phenotype especially in later generations. Significantly, midgut-specific IR-RNA expression continued to be detectable in a GDLS genetic background after introgression of the Carb109F and Carb109M transgenes (Fig. S2C).

### IR-RNA processing in Carb109M by the endogenous RNAi machinery

**Figure 2:**
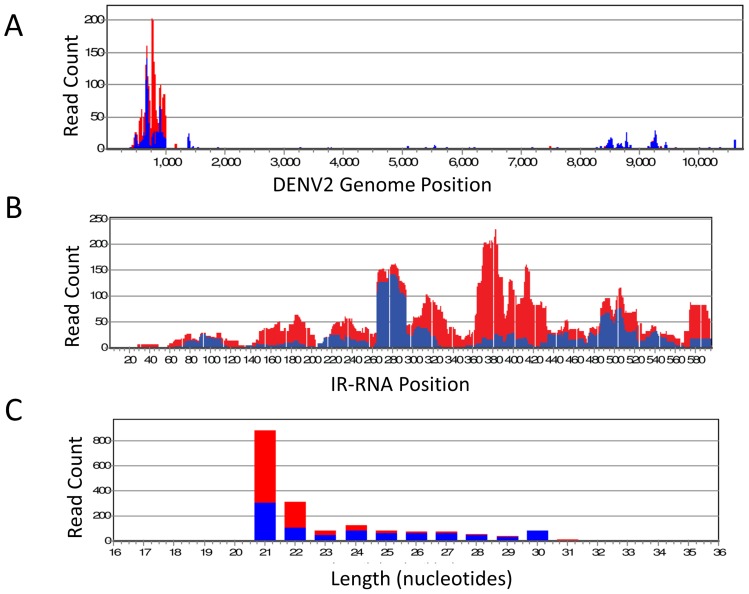
NextGen small RNA sequencing to reveal processing of the IR-RNA into siRNAs in Carb109M mosquitoes. (A) Alignment of small RNA sequences derived from the IR-RNA to the DENV2-Jamaica1409 genome (10,723 nucleotides). (B) Alignment of small RNA sequences to the 568 nt region of DENV2-Jamaica1409 RNA targeted by the IR (Mnp) effector gene. (C) Size distribution of small RNA reads. Y-axis: Read Count  =  number of small RNAs detected by NextGen sequencing. X-axis: Distribution of small RNA sequences mapped to the DENV2-Jamaica1409 genome. Red bars, negative-sense small RNAs; blue bars, positive-sense small RNAs.

We performed NextGen sequencing with Carb109M to detect size and specificity of small RNAs generated from the IR-RNA of the effector gene. NextGen sequencing of size-fractionated, total RNA from Carb109M midguts at 20 h post-bloodmeal was performed to determine whether the expressed IR-RNA was processed as a double-stranded RNA by the vector's RNAi machinery. The DENV2-specific small RNA profile revealed that the IR RNA was processed into siRNAs within hours of Carb109M mosquitoes ingesting a bloodmeal containing no virus (Fig. 2). Alignment of the small RNA sequences to the whole genome of DENV2-Jamaica1409 revealed a cluster of small RNAs from nucleotide positions nt 450–1000 mapping to the prM-M coding region (Fig. 2A). Alignment of the small RNA sequences to the 568 nt region of DENV2-Jamaica1409 RNA targeted by the IR effector sequence had significant overlap with sequences at nucleotide positions corresponding to the prM-M coding region (Fig. 2B). Small regions of complementarity were concentrated at nucleotide positions 260–440, with representation reaching up to 220 read counts per small RNA (Fig. 2B). Size-distribution plotting of small RNAs showed that the predominant size was 21 nucleotides; the hallmark of the antiviral siRNA pathway (Fig. 2C). Interestingly, there was a bias towards siRNAs in antisense orientation even though it was expected that RNAi degradation of the IR effector would have produced equal proportions of sense- and antisense-oriented siRNAs.

### Characterization of transgene integration in Carb109M and Carb109F mosquitoes

**Figure 3:**
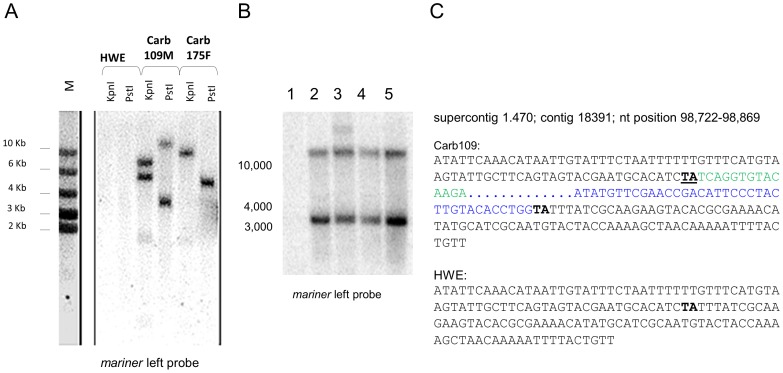
Characterization of transgene integration in Carb109 mosquitoes. (A) Southern blot analysis to detect transgene integration sites among transformed *Ae. aegypti*. (B) Southern blot analysis to detect transgene integration sites in Carb109M, Carb109F, Carb109M/GDLS.BC_6_, and Carb109F/GDLS.BC_6_. Lane 1: HWE; lane 2: Carb109F G_9_; lane 3: Carb109M G_9_; lane 4: Carb109F/GDLS.BC_6_; lane 5: Carb109M/GDLS.BC_6_. Total DNA was digested with *Pst*I. (C) Physical mapping of a transgene integration site in Carb109M mosquitoes. In bold: *mariner Mos1* TA target sequence motif; in bold and underlined: duplication of the TA target sequence as a consequence of *mariner Mos1* integration; highlighted in green and blue: partial sequences of the left and right arms of the TE.

Southern blot analysis with a [α-^32^P]dCTP DNA probe derived from *mariner Mos1* left arm sequence (Fig. 1A) indicated that Carb109M had two transgene integrations (Fig. 3A). If a single integration event occurred then a *Kpn*I restriction endonuclease digestion of Carb109M DNA should detect a single junction fragment (1389 bp of transgene specific DNA plus vector DNA). A *Pst*I digestion of Carb109M DNA should also detect a single junction fragment (1,476 bp of the transgene plus vector DNA). Instead, *Kpn*I digestion and Southern blot analysis of Carb109M detected two junction fragments (∼5000 bp and ∼6000 bp) supporting the conclusion that the transgene was integrated at two loci in Carb109M. *Pst*I digestion and Southern blot analysis of Carb109M confirmed this finding by detecting two junction fragments (∼3500 bp and >10,000 bp). In contrast, Southern blot analysis of Carb175F DNA showed integration of the transgene at a single locus (Fig. 3A). The transgene integration patterns of Carb109M and Carb109F were identical by Southern blot analysis and maintained the same pattern after introgression of the transgene into the GDLS genetic background (Fig. 3B).

Genome walking was performed with Carb109M and identified one of the two transgene integration loci in the 3′ UTR of gene AAEL010318 (VectorBase supercontig 1.470), encoding a polyadenylate binding protein, mapping terminally at 70 cM of chromosome 3, and in physical division 3q4.4 as determined by Fluorescent *in situ* Hybridization (FISH) [46]. The integration of the TE occurred at a canonical TA recognition sequence motif resulting in target-site duplication. The second transgene integrated in a repetitive (>50 copies) sequence motif and was atypical by including a 920 bp portion of the *pMos1* plasmid backbone extending from the left arm of the TE (data not shown). The physical integration pattern of the second integration event extending from the right arm of the TE was not detectable by genome walking. The site of integration(s) in Carb109F has not been analyzed by genome walking.

### DENV2, DENV3, and CHIKV challenge of Carb109M mosquitoes

**Figure 4:**
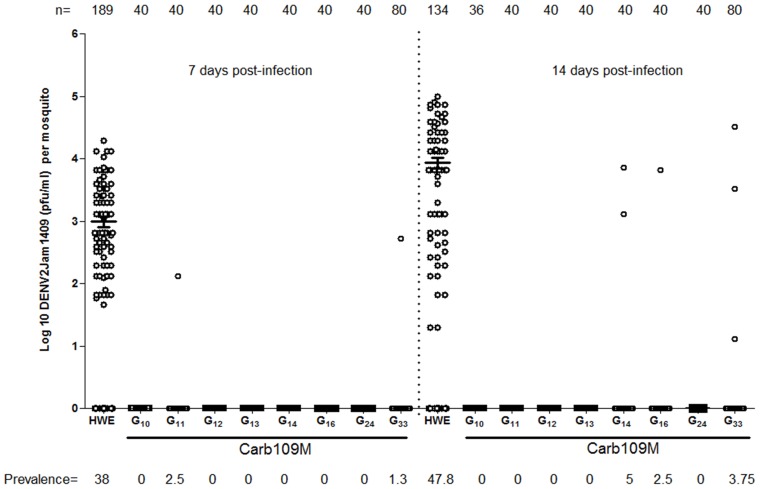
Analysis of Carb109M DENV2-resistance phenotype over 33 intercrossed generations. HWE (control) and Carb109M mosquitoes received bloodmeals containing 10^6^ pfu/ml DENV2-Jamaica1409. Virus titers in the mosquitoes were assessed at 7 and 14 dpi. Each data point represents the virus titer of a single female. Mean values and standard errors are indicated.

**Figure 5:**
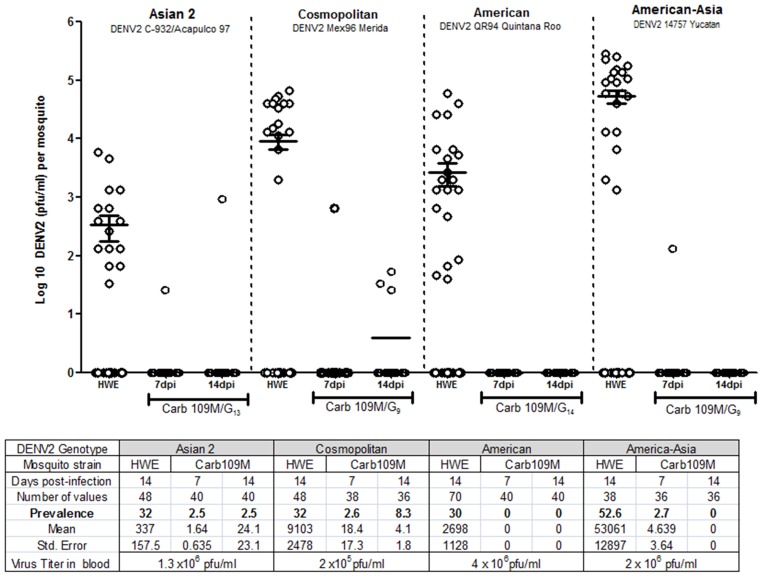
Carb109M resistance phenotype when challenged with different Mexican DENV2 genotypes. HWE (control) and Carb109M (G_9_, G_13_, G_14_) mosquitoes were infected with DENV2 C-932/Acapulco 97, Mex96 Merida, QR94 Quintana Roo, and 14757 Yucatan, representing the Asian 2, Cosmopolitan, American, and Asian-American genotypes, respectively. Mosquitoes received DENV2-containing bloodmeals with the following titers: 1.3×10^6^ pfu/ml (C-932/Acapulco 97), 2.0×10^5^ pfu/ml (Mex96), 4.0×10^6^ pfu/ml (QR94), and 2.0×10^6^ (14757). Virus titers in the mosquitoes were assessed at 7 and 14 dpi. Each data point represents the virus titer of a single female. Mean values and standard errors are indicated.

Carb109M (G_10_–G_33_) mosquitoes were infected with ∼10^6^ pfu/ml of DENV2-Jamaica1409 via artificial bloodfeeding. The prevalence at 7 and 14 dpi was only 0.5% (2/360) and 1.7% (6/356), respectively (Fig. 4). The HWE control had >35% prevalence at either time point with mean DENV2 titers of 1000 and 9,000 pfu/ml at 7 and 14 days dpi, respectively. The absence of DENV2 titers in Carb109M mosquitoes demonstrates clearly that the refractory phenotype was stable over multiple generations even though occasionally, single Carb109M females had detectable DENV2 titers (Fig. 4). Carb109M mosquitoes also were resistant to different Mexican DENV2 isolates, C-932/Acapulco 97, Mex96 Merida, QR94, and 14757 Yucatan, representing Asian 2, Cosmopolitan, American, and American-Asian genotypes, respectively (Fig. 5). The sequence of the IR gene derived from the DENV2-Jamaica1409 genome had >86% minimal nucleotide identity with isolates representing the four DENV2 genotypes isolated in Mexico (Fig. S3). Mean DENV2 titers in HWE at 14 dpi were 340 pfu/ml for C-932/Acapulco 97 (32% prevalence), 9100 pfu/ml for Mex96 (32% prevalence), 2700 pfu/ml for QR94 (30% prevalence), and 53,000 pfu/ml for 14757 (52.6% prevalence). Prevalence in Carb109M females never exceeded 8.3% as shown for DENV2 Mex96.

Carb109M mosquitoes challenged with DENV3 or with an unrelated arbovirus (CHIKV) did not show a refractory phenotype to these viruses (Fig. S4 and S5). At 7 days dpi, HWE mosquitoes had mean DENV3 6889/QR-MX/97 titers of 1300 pfu/ml and Carb109M had mean virus titers of 750 pfu/mL (Fig. S4). There is <60 % sequence identity in the 568 nt target region between the genome of DENV3 6889/QR-MX/97 and that of DENV2-Jamaica1409, the source of the Mnp effector gene sequence. At 7 dpi, DENV3 prevalence was similar between HWE, Carb109M, Carb175F, and the Carb52 control. However, at 14 days dpi, DENV3 prevalence was significantly higher for HWE (80%) than for Carb109M or Carb175F (55%) (Fisher's exact test, p = 0.0307). Infecting the same mosquito lines with CHIKV 37997 showed no statistical differences in prevalence (90–100%) (Fig. S5). All these data confirm the sequence-dependent nature of the resistance mechanism in Carb109M mosquitoes.

### Refractory phenotype of Carb109/GDLS.BC_5_ mosquitoes for DENV2

**Figure 6:**
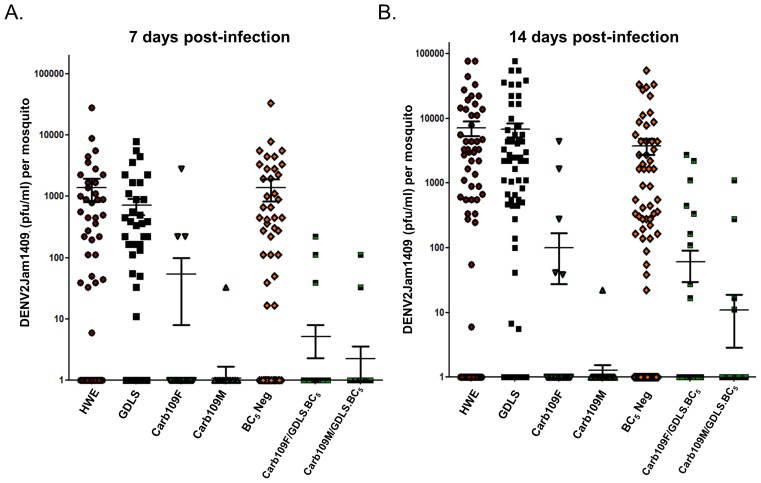
Analysis of resistance to DENV2 infection in Carb109M/GDLS.BC_5_ and Carb109F/GDLS.BC_5_ mosquitoes. The Carb109 transgene was introgressed via five consecutive backcrosses into GDLS. HWE (control), GDLS (control), Carb109M, Carb109F, Carb109M/GDLS.BC_5_, Carb109F/GDLS.BC_5_ were challenged with DENV2-Jamaica1409 (titer in the bloodmeal: 10^6^ pfu/ml). BC_5_-neg mosquitoes were used as additional controls and represented mosquitoes not positive for the eye-marker after five backcrosses. Virus titers of mosquitoes were assessed at (A) 7 and (B) 14 dpi. Each data point represents the virus titer of a single female. Mean values and standard errors are indicated.

**Figure 7:**
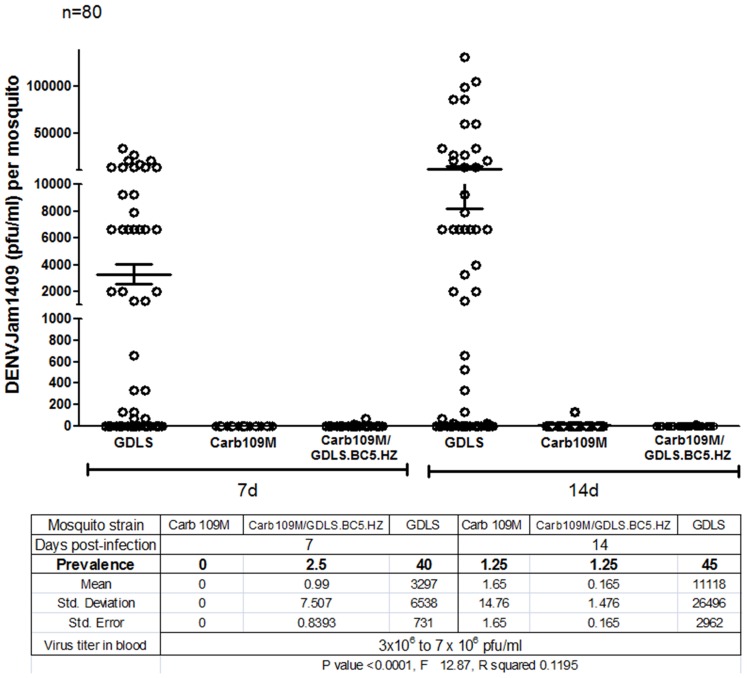
Analysis of resistance to DENV2 infection of homozygous Carb109M/GDLS.BC_5_.HZ mosquitoes. GDLS, Carb109M, Carb109M/GDLS.BC_5_.HZ mosquitoes were challenged with DENV2-Jamaica1409 (titer in the bloodmeal: >10^6^ pfu/ml). Virus titers of mosquitoes were assessed at 7 and 14 dpi. Each data point represents the virus titer of a single female. Mean values and standard errors are indicated.

We assessed whether the DENV2 refractory phenotype was maintained after introgression of the Carb109 transgene into a diverse genetic background. The GDLS strain is highly susceptible to DENV2-Jamaica1409 at 7 and 14 dpi (Fig. 6). Following five consecutive backcrosses to GDLS, Carb109F/GDLS.BC_5_ and Carb109M/GDLS.BC_5_ mosquitoes remained highly refractory to DENV2 infection at both time points, similar to line Carb109M, although Carb109F/GDLS.BC_5_ tended to be more susceptible to the virus at 14 dpi than Carb109M/GDLS.BC_5_ (Fig. 6). However, Carb109F/GDLS/BC_5_ and Carb109M/GDLS.BC_5_ mosquitoes had no significant difference with regard to DENV2 resistance. HWE, GDLS and BC_5_ Neg (negative for the EGFP marker) mosquitoes showed a significantly higher prevalence of DENV2 (59.6–67.5%) than Carb109M, Carb109F, Carb109M/GDLS.BC_5_, and Carb109F/GDLS.BC_5_ mosquitoes (1.8–7.4%) at 7 and 14 dpi (Fig. 6). Furthermore, as stated earlier both transgene integrations were detected by Southern blot analysis from Carb109M/GDLS.BC_5_ and Carb109F/GDLS.BC_5_ mosquitoes (Fig. 3B). Finally, homozygous line Carb109M/GDLS.BC_5_.HZ was highly refractory to DENV2 with only 3/160 mosquitoes having detectable virus titers (<200 pfu/mL) at 7 or 14 dpi (Fig. 7).

### Fitness of the Carb109F and Carb109M transgenes backcrossed into the GDLS background

**Figure 8:**
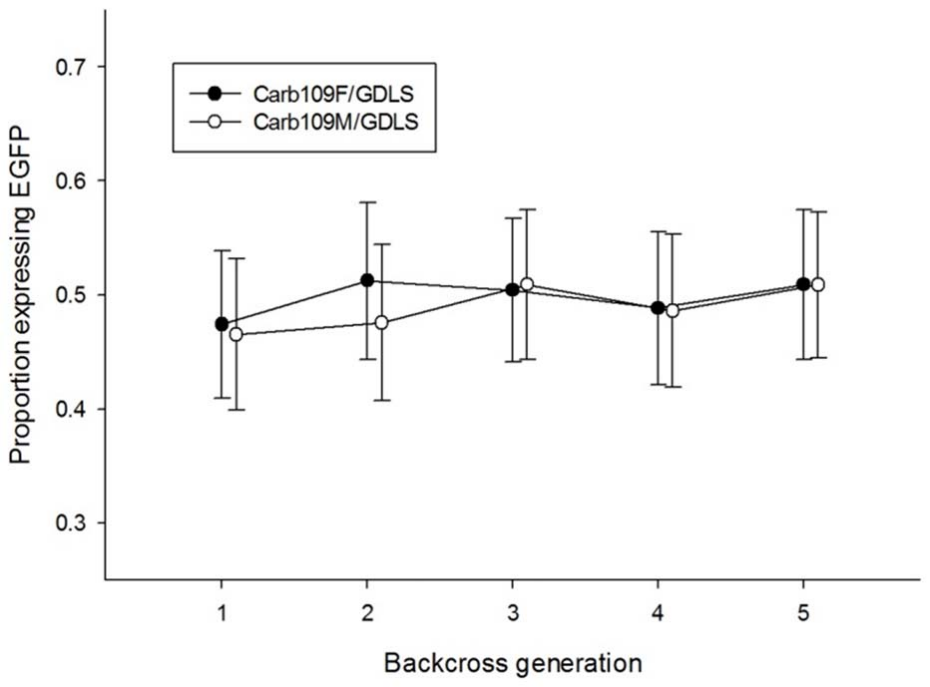
Proportions of EGFP-expressing Carb109M and Carb109F larvae during five consecutive backcrosses to GDLS. Carb109F and Carb109M were crossed reciprocally with GDLS mosquitoes. Wild-type individuals were culled and remaining EGFP expressing transgenic individuals (Carb109F/GDLS.BC_1_ and Carb109M/GDLS.BC_1_) were backcrossed further to GDLS for four additional generations. Proportions of EGFP-expressing larvae in each of the five backcross generations in Carb109F/GDLS (filled circles) and Carb109M/GDLS (open circles) are shown. Proportions were compared by estimating the 95% HDI (error bars) with WinBUGS and the Credible Intervals for Proportions script [44].

The 95% HDI for the proportion of larvae expressing EGFP overlapped among all of the five backcross generations of both Carb109F/GDLS and Carb109M/GDLS (Fig. 8). Furthermore, all 95% HDI contained the expected 0.5 proportion expected for heterozygotes.

**Figure 9:**
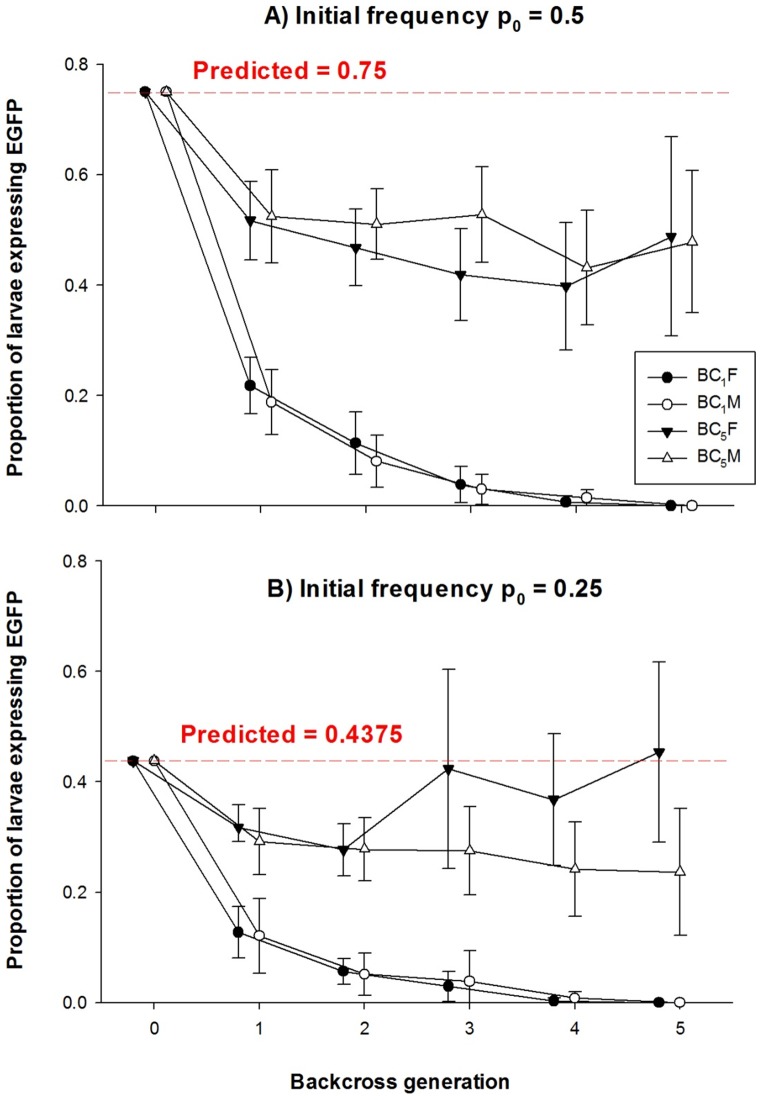
Transgenic allele frequencies among Carb109/GDLS backcrossed mosquitoes over five generations (F_1_–F_5_) in absence of selection for the transgenic phenotype. Initial frequencies (p_0_) of the Carb109 transgene were either (A) 0.5 (transgenic heterozygote x transgenic heterozygote) for Carb109F/GDLS.BC_1_, Carb109M/GDLS.BC_1_, Carb109F/GDLS.BC_5_, and Carb109M/GDLS.BC_5_ or (B) 0.25 (transgenic heterozygote x GDLS) for Carb109F/GDLS.BC_1_, Carb109M/GDLS.BC_1_, Carb109F/GDLS.BC_5_, and Carb109M/GDLS.BC_5_. Fifteen lines were established for each of the eight experiments. Proportions of EGFP-expressing offspring were estimated by examining ∼150 larvae from each of the 15 lines over five successive generations (F_1_–F_5_) of inter-breeding without selection for the transgenic phenotype. Bars around mean proportions represent Bayesian 95% Highest Density Intervals (95% HDI). Proportions showing non-overlapping 95% HDI are credibly different.

The frequencies of EGFP-expressing larvae were compared among Carb109F/GDLS.BC_1_, Carb109M/GDLS.BC_1_, Carb109F/GDLS.BC_5_ and Carb109M/GDLS.BC_5_ at starting frequencies of 0.25 and 0.5. Frequencies were measured over five generations without selection for EGFP (Fig. 9A, B). In all BC_1_ lines, the proportion of larvae expressing EGFP declined rapidly and reached zero by the fifth generation. This occurred whether p_0_ was 0.5 (Fig. 9A) or 0.25 (Fig. 9B).

Estimated fitness coefficients (Table 2) among these four BC_1_ variants (Carb109F/GDLS.BC_1_, p_0_ = 0.25, Carb109M/GDLS.BC_1_, p_0_ = 0.25, Carb109F/GDLS.BC_1_, p_0_ = 0.5, Carb109M/GDLS.BC_1_, p_0_ = 0.5) were W_AA_  = 0.01 in all four experiments while W_Aa_ varied from 0.11–0.21 and W_aa_ varied between 0.94–1.00, respectively. This supports the conclusion that either the transgene has a dominant fitness load or that a deleterious allele(s) was linked (in *cis*) to the transgene insertion site. In contrast, the proportions of EGFP-expressing larvae in Carb109M/GDLS.BC_5_ and Carb109F/GDLS.BC_5_ when initiated at p_0_ = 0.5 were 0.4881 and 0.4782 in F_5_. However, the 95% HDI surrounding these frequencies did not contain the predicted 0.75 allele frequency. Again the predicted fitness coefficient for transgene homozygotes was W_AA_  = 0.01 in all four experiments while W_Aa_ varied from 0.94–1.00, which actually exceeded W_aa_ (0.65–0.93) in the four BC_5_ experiments. The proportions of EGFP expressing larvae in Carb109F/GDLS.BC_5_ and Carb109M/GDLS.BC_5_ initiated at p_0_ = 0.25 (Fig. 9B) were 0.4538 and 0.2365 in F_5_. However, only the 95% HDI surrounding frequencies in Carb109F/GDLS.BC_5_ (BC_5_F in Fig. 9) contained the predicted 0.4375 frequency. The fact that the predicted fitness coefficient for transgene homozygotes was very low (W_AA_ = 0.01), whereas fitness coefficients for transgene heterozygotes (W_Aa_ = 0.94–0.95) and those for wildtypes (W_aa_ = 0.81–0.93) were significantly higher supports the interpretation that the wild-type allele had a dominant positive effect on fitness. Thus, five generations of backcrossing did not change the fitness of transgene homozygotes but greatly improved the fitness of transgene heterozygotes.

**Table 2:** Fitness coefficients estimated by Maximum Likelihood search for coefficients most consistent with the observed frequencies of EGFP expressing larvae following A) backcrossing for one or five generations without selection for the transgene or B) backcrossing for five generations with selection for the transgene after each backcrossing step.

Backcrosses-Replicate-Initial transgene frequency	Transgenic homozygote fitness (WAA)	Transgenic heterozygote fitness (WAa)	Transgenic Homozygote/Heterozygote (WAA/Waa)	Wild-type homozygote (Waa)
A) Backcrossing				
BC1-ReplicateF-p0 = 0.25	0.01	0.21	0.05	0.94
BC1-ReplicateF-p0 = 0.5	0.01	0.13	0.08	0.94
BC1-ReplicateM-p0 = 0.25	0.01	0.21	0.05	1.00
BC1-ReplicateM-p0 = 0.5	0.01	0.11	0.09	0.98
BC5-ReplicateF-p0 = 0.25	0.01	0.95	0.01	0.81
BC5-ReplicateF-p0 = 0.5	0.01	0.95	0.01	0.78
BC5-ReplicateM-p0 = 0.25	0.01	0.94	0.01	0.93
BC5-ReplicateM-p0 = 0.5	0.01	1.00	0.01	0.65
B) Selection				
Selection-F.1	0.32	1.00	0.32	0.00
Selection-F.2	0.51	0.91	0.56	0.00
Selection-F.3	0.50	0.68	0.74	0.00
Selection-M.1	0.74	0.81	0.91	0.00
Selection-M.2	0.77	0.75	1.03	0.00
Selection-M.3	0.72	0.86	.84	0.00

### Fitness of the Carb109F and Carb109M transgenes during selection

**Figure 10:**
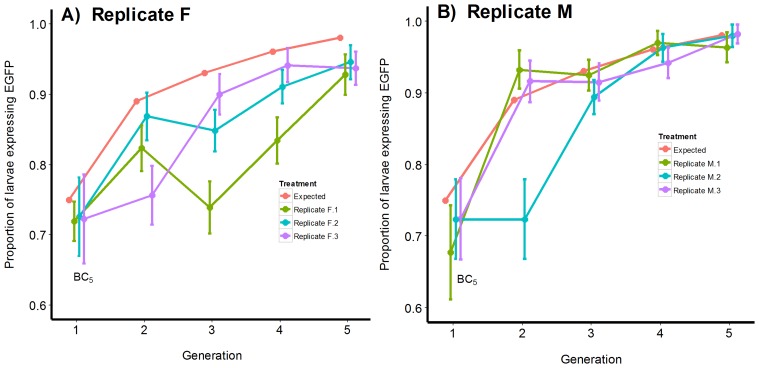
Comparison of observed transgenic allele frequencies over five generations (F_1_–F_5_) of Carb109F/GDLS.BC_5_ and Carb109M/GDLS.BC_5_. Three replicate crosses (F.1, F.2, F.3) between Carb109F/GDLS.BC5 heterozygotes (200 individuals in each cross) and three replicate crosses (M.1, M.2, M.3) between Carb109M/GDLS.BC5 heterozygotes (200 individuals/cross) were performed in separate cages. All resulting wild-type larvae were culled. Remaining transgenic individuals were allowed to inter-mate. This procedure was followed for five generations (F_1_–F_5_). Results were compared to values expected under Fisher's Selection Model. (A) Observed frequencies of EGFP- expressing Carb109F/GDLS.BC_5_ larvae of replicates F.1, F.2, F.3 compared to values predicted under Fisher's Selection model (redline). (B) Observed frequencies of EGFP-expressing Carb109M/GDLS.BC_5_ larvae of replicates M.1, M.2, M.3 in comparison to values predicted under Fisher's Selection model (redline). Proportions were compared by estimating the 95% HDI (error bars) with WinBUGS and the Credible Intervals for Proportions script [44].

Selection was applied to three lines each of Carb109F/GDLS.BC_5_ and Carb109M/GDLS.BC_5_ heterozygotes. In each generation wild-type larvae were culled and mosquitoes were allowed to inter-mate. This was repeated over four consecutive generations (Fig. 10). Observed values were compared with values predicted from equation 4. Replicate F.1 remained below predictions of Fisher's model from generations 2 through 5 (Fig. 10A). Replicate F.2 reached predictions for generation 2 but then remained lower than predictions from generations 3 through 5. Replicate F.3 reached model predictions for generation 4 but was lower than predicted in generation 5. The 95% HDI surrounding proportions of EGFP-expressing larvae in generation 5 for all three F replicates did not cover the expected 0.9822 allele frequency.

Replicate M.1 started below the expected 0.75 value but exceeded predictions in generation 2 and overlapped predictions in generations 3–5 (Fig. 10B). Replicate M.2 fell below predictions for generation 2 and 3 but reached predictions in generations 4 and 5. Only replicate M.3 tracked model predictions in all generations. The 95% HDI surrounding proportions of EGFP-expressing larvae in generation 5 for all three M replicates contained the expected 0.9822 allele frequency.

Among the three F replicated lines the fitness coefficients of transgene homozygotes (W_AA_) ranged from 0.32–0.51, a dramatic improvement over the BC_5_ values of W_AA_ = 0.01 (Table 2). Fitness of transgene heterozygotes (W_Aa_ = 0.68–1.00) on the other hand overlapped the BC_5_ values (W_Aa_ = 0.95). Among the three M replicates the fitness coefficients of transgene homozygotes (W_AA_) ranged from 0.72–0.77, far exceeding the BC_5_ values of W_AA_ = 0.01 (Table 2). However, fitness of M replicate transgene heterozygotes (W_Aa_ = 0.75–0.86) was lower than the BC_5_ values (W_Aa_ = 0.94–1.00). Family-based selection was conducted in 30 families in each of replicates M.1–M.3 and F.1–F.3. However, we were successful in breeding only one homozygous family (Carb109M/GDLS.BC_5_.HZ) by family-based selection on Replicate M.3.

## Discussion

We showed in an earlier proof of principle study that *Ae. aegypti* can be genetically modified for DENV resistance by establishing an RNAi-based infection barrier in the midgut [16]. However, Carb77 eventually lost its refractory phenotype to DENV2 before we completed introgression of the transgene into a more genetically diverse vector population. Here, we have developed and characterized a panel of new transgenic *Ae. aegypti* lines and have identified additional lines refractory to DENV2 and suitable for introgression studies. In previous, unpublished experiments (Dr. Bill Black, CSU) a transgene was introgressed into GDLS and after 5 backcrosses, 4 generations of selection and construction of the final strain from only 34 families there was only a very small decrease in genetic diversity (a drop from 0.257 to 0.251). Allele frequencies did not change at four of 10 loci. Given the very large numbers of genomes involved in these experiments, we considered it unlikely that these shifts arose via genetic drift but rather reflect laboratory adaptations. Our conclusion was that the transgene introgression strategy into GDLS was effective in maintaining genetic diversity. We report the development of a mosquito line (Carb109M/GDLS.BC_5_.HZ) expressing an antiviral effector RNA in the midgut, and displaying a refractory DENV2 phenotype. This line should be suitable for analyzing and modeling transgene spread between mosquito populations using strategies such as Reduce and Replace [47], [48].

The development and characterization of one transgenic line, Carb109M, resulted in five key findings. First, the transgenic genotype and DENV2 refractory phenotype of Carb109M mosquitoes in a HWE genetic background has remained stable over 33 generations. Second, the DENV2 refractory phenotype remained stable after five consecutive backcrosses into a genetically diverse background followed by four generations of selection and two generations of family-based selection. Third, backcrossing the transgene dramatically improved the fitness of the transgene heterozygotes from W_Aa_ = 0.11–0.21 after one backcross to W_Aa_ = 0.94–1.00 after five generations of backcrossing. However, the fitness of transgene homozygotes remained at W_AA_ = 0.01 after five generations of backcrossing. Fourth, four generations of selection improved transgene homozygous fitness from W_AA_ = 0.01 to 0.32–0.51 in the F replicates and from W_AA_ = 0.01 to 0.72–0.77 in the M replicates. As discussed below, this is consistent with the presence of a deleterious recessive allele linked with the transgene but which gradually became unlinked with recombination. Fifth, family based selection involving 180 families yielded only one homozygous line (Carb109M/GDLS.BC_5_).HZ line. This line was highly refractory to DENV2.

We observed a few Carb109M/GDLS.BC_5_.HZ individuals infected with DENV2. This also was observed with Carb77 mosquitoes and may be a consequence of using artificial bloodmeals, which occasionally might lead to a leaky midgut phenomenon [49]. A concern is the possibility that the antiviral IR-RNA and siRNAs from the transgene promote selection for viral escape mutants, capable of evading our RNAi-based strategy. However, our results show that nucleotide sequence diversity over the 568 nt target region of the DENV2-specific RNAi trigger can reach 14% without compromising the sequence-dependent RNAi degradation mechanism. This presumably occurs because a number of siRNAs derived from the 568 bp effector RNA have exact matches with the sequence of each of the DENV2 genotypes. At this point we consider it unlikely that the effector RNA of Carb109M could select for a viable DENV2 mutant that has >14% sequence diversity in the prM-M encoding region of its viral RNA to escape the RNAi response in the transgenic mosquitoes. Nevertheless, the possible occurrence of RNAi escape mutants will be addressed in future experiments.

The transgene integration pattern in Carb109M may account for the strong DENV2 resistance phenotype. In contrast to Carb77, which had a single transgene integration event, Carb109M and Carb109F mosquitoes appear to have two independent insertions, which potentially result in an increased dose of the IR-RNA effector. If the quantity of IR-RNA produced in a cell is a rate-limiting factor determining the efficiency of the RNAi response, higher IR-RNA expression would degrade a proportionally higher number of target RNAs at any given time. The site of transgene integration cannot be targeted using a TE such as *mariner Mos1* as the insertion vector, and this may lead to position-effects impacting transgene expression. A reason for the loss of the resistance phenotype in Carb77 mosquitoes could have been chromatin re-arrangements that led to silencing of the IR effector gene expression while leaving the transgene intact at the DNA level [33], [35]. In contrast, Carb109M had an integration of the transgene in the 3′UTR of the polyadenylate binding protein gene (AAEL010318) and another in a highly repetitive DNA motif. At this point we cannot explain why the integration pattern of the transgene in Carb109M mosquitoes produced more stable effector RNA expression than that observed in Carb77 mosquitoes. Two strategies can be employed to avoid transgene position effects in arthropods. One strategy is the use of a site-specific recombination system such as φ C31 [39], [50]. Another strategy is the use of chromatin insulators such as those derived from the Drosophila *gypsy* retrotransposon [51], [52], which have not currently been used in *Ae. aegypti*.

We propose that the initial rate and pattern of decline in the frequency of EGFP-expressing BC_1_ and BC_5_ larvae maintained without selection is consistent with the presence of deleterious or lethal recessive allele (or allele) originating from the HWE chromosome into which the transgene was originally inserted. This would explain why five generations of backcrossing increased the fitness of transgene heterozygotes but not homozygotes. Only a single wild-type allele would have been sufficient to cover the deleterious recessive allele linked to the transgene in heterozygotes. It is unlikely that the transgene itself was under negative selection due, for example, to overexpression of the EGFP reporter or the transgene disrupting expression of the polyadenylate-binding protein gene into which it is inserted. The insertion site is 679 bp 3′ from the stop codon in the 3′UTR. Further, if the transgene itself were under strong negative selection we would not have succeeded in breeding a homozygous Carb109M/GDLS.BC_5_.HZ line. Nor would backcrossing and selection have improved the fitness of transgene homozygotes and heterozygotes (Table 2).

Several studies have documented deleterious and lethal recessives in inbred lines of *Ae. aegypti* and several loci of this type have been mapped [53]–[55]. These are maintained as heterozygotes through balancing selection. Through rare recombination during backcrossing and selection, the deleterious allele of HWE origin might have recombined once or a few times with wild type alleles from the GDLS and thus become disassociated with the transgene. An additional factor may be that the GDLS strain was generally more fit than the HWE strain, which was inbred initially to generate homozygous white-eye mosquitoes and then maintained for over 20 years in the laboratory. In this case, a beneficial gene originating from the GDLS background would have become linked to the Carb109 transgene.

If this is the case then we believe that crossover events between wild-type GDLS alleles and/or deleterious alleles from the HWE strain were rare (i.e. the transgene and deleterious alleles were closely linked). This would explain why eventually only one of the six selected lines responded to selection in the predicted manner. It also would explain the discrepancy between the final allele frequencies in the BC_5_ F and M replicate strains (Fig. 9B). Residual linkage between the transgene and deleterious allele would also explain why family based selection on 180 families yielded only one homozygous Carb109M/GDLS.BC_5_.HZ line.

Regardless of the mechanism, the results of the backcross and selection experiments emphasize the importance of outcrossing transgenes into genetically-diverse, recently-colonized strains before attempting to assess fitness loads imposed by the transgene and certainly before driving transgenes into natural or laboratory-maintained populations. Two additional components will be necessary to successfully implement a population replacement-based DENV control strategy in the field. (1) Design of an effector gene that targets and destroys simultaneously the genomes of all four DENV serotypes. Earlier attempts to design a tetravalent IR effector gene targeting the NS5 region of all four DENVs failed due to technical problems, although we are confident this problem can be overcome. (2) The anti-DENV effector gene may require linkage to an *Ae. aegypti*-specific gene drive system to enable fixation of the transgene in the target population. The Reduce and Replace strategies and killer-rescue based gene drive systems such as Medea are currently under development in *Ae. aegypti* [47], [48], [56]–[58].

## Supporting Information

Figure S1 **DENV2-Jamaica1409 challenge of transgenic lines Carb1M, Carb1F, Carb22M, Carb96F, Carb109F, Carb109M, Carb175F, Carb194M, and Carb203F.** HWE mosquitoes and line Carb52 (expressing a fluorescent reporter in midgut tissue) were used as control. The DENV2 titers in the bloodmeals ranged from 1.5×10^6^ to 1.6×10^7^ plaque forming units (pfu)/ml. (A) DENV2 infections at 7 dpi. (B) DENV2 infections at 14 dpi. Each data point represents the virus titer of a single female. Mean values and standard errors are indicated.
(TIF)

Figure S2 **Detection of DENV2-specific IR-RNA isolated from midguts of bloodfed Carb109M, Carb109F, and Carb175F mosquitoes.** (A) Detection of IR RNA in midguts of bloodfed females of lines Carb109F and Carb109M. HWE and Carb52 mosquitoes were used as controls. (B) The same effector RNA was detected weakly in midguts of bloodfed Carb175F females. Blots were exposed for 72 h using Carb109M RNA as control. (C) Detection of IR effector RNA at 20 h post-bloodmeal (pbm) in midguts of Carb109F/GDLS.BC_6_ and Carb109M/GDLS.BC_6_ (top). The ethidium-bromide stained gel is shown as a loading control (bottom). Blots were hybridized a probe corresponding to the prM-M encoding cDNA of DENV2. Arrow indicates a 500 nt RNA marker.
(TIF)

Figure S3 **Nucleotide sequence alignment of the 568 nt prM-M encoding region of different DENV2 isolates from Mexico.** DENV2 isolates C-932/Acapulco 97, Jam1409, Mex96 Merida, QR94 Quintana Roo, and 14757 Yucatan represent four different DENV2 genotypes targeted by the IR-RNA. RNAi is a homology-dependent antiviral pathway and Carb109M displayed a refractory phenotype to the four viruses.
(TIF)

Figure S4 **DENV3 challenge of Carb109M mosquitoes.** HWE (control), Carb175F, Carb109M, and Carb52 (transgenic control expressing a fluorescent reporter in midgut tissue) mosquitoes received an oral bloodmeal containing 2.3×10^6^ pfu/ml DENV3-6889/QR-MX/97. Virus titers of mosquitoes were assessed at 7 and 14 dpi. Each data point represents the virus titer of a single female. Mean values and standard errors are indicated.
(TIF)

Figure S5 **CHIKV challenge of Carb109M mosquitoes.** HWE (control), Carb175F, Carb109M, and Carb52 (transgenic control expressing a fluorescent reporter in midgut tissue) mosquitoes received an oral bloodmeal containing 8.13×10^7^ pfu/ml CHIKV 37997. Virus titers of mosquitoes were assessed at 5 and 12 dpi. Each data point represents the virus titer of a single female. Mean values and standard errors are indicated.
(TIF)
