# Supplementary figures and images for: Fitness Impact and Stability of a Transgene Conferring Resistance to Dengue-2 Virus following Introgression into a Genetically Diverse Aedes aegypti Strain
Source: PLoS Negl Trop Dis. 2014 May 8;8(5):e2833. doi: 10.1371/journal.pntd.0002833 (PMC4014415; doi:10.1371/journal.pntd.0002833)

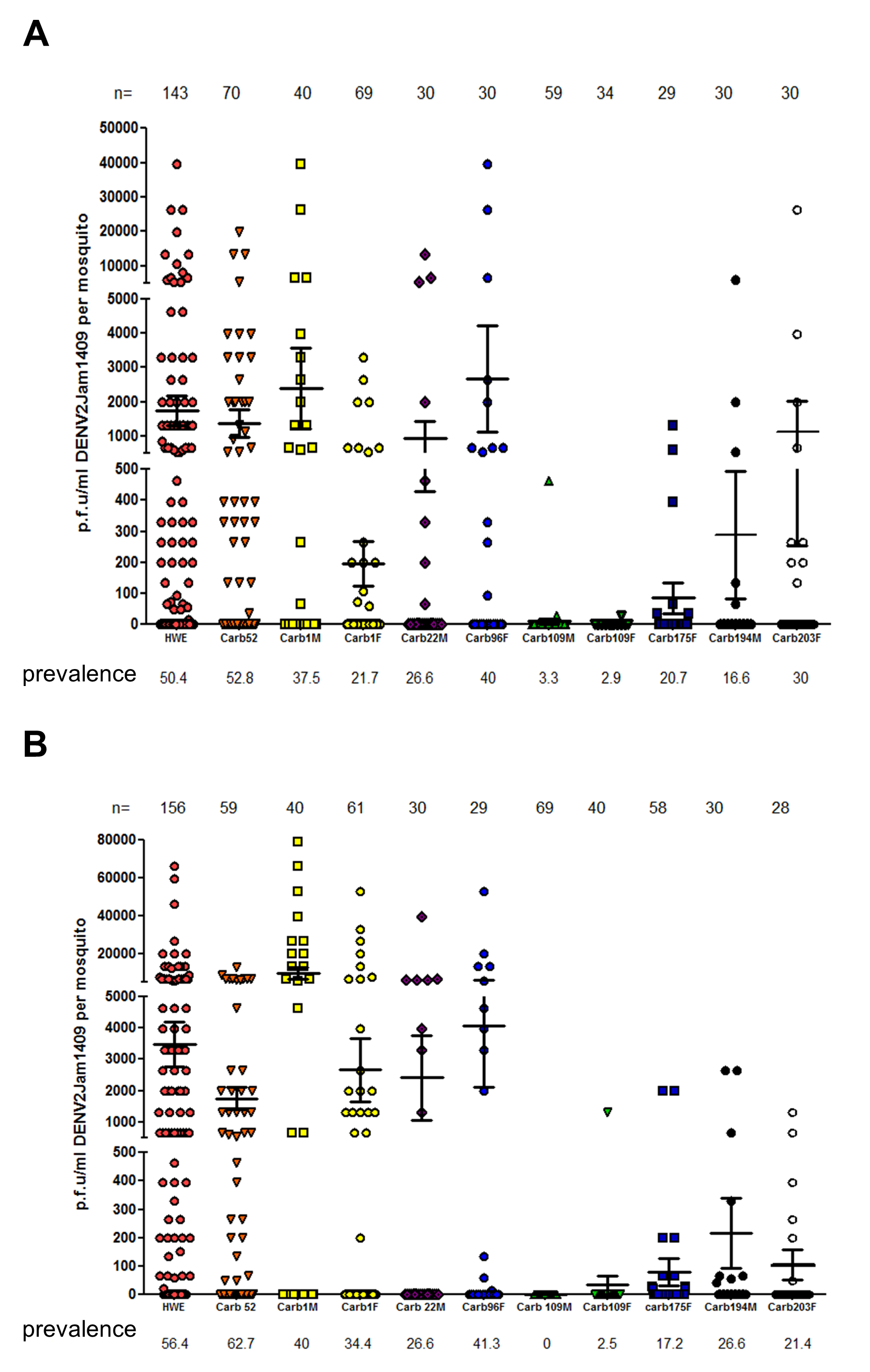

Supplement: Figure S1 — DENV2-Jamaica1409 challenge of transgenic lines Carb1M, Carb1F, Carb22M, Carb96F, Carb109F, Carb109M, Carb175F, Carb194M, and Carb203F. HWE mosquitoes and line Carb52 (expressing a fluorescent reporter in midgut tissue) were used as control. The DENV2 titers in the bloodmeals ranged from 1.5×10^6^ to 1.6×10^7^ plaque forming units (pfu)/ml. (A) DENV2 infections at 7 dpi. (B) DENV2 infections at 14 dpi. Each data point represents the virus titer of a single female. Mean values and standard errors are indicated. (TIF) [file pntd.0002833.s001.tif]

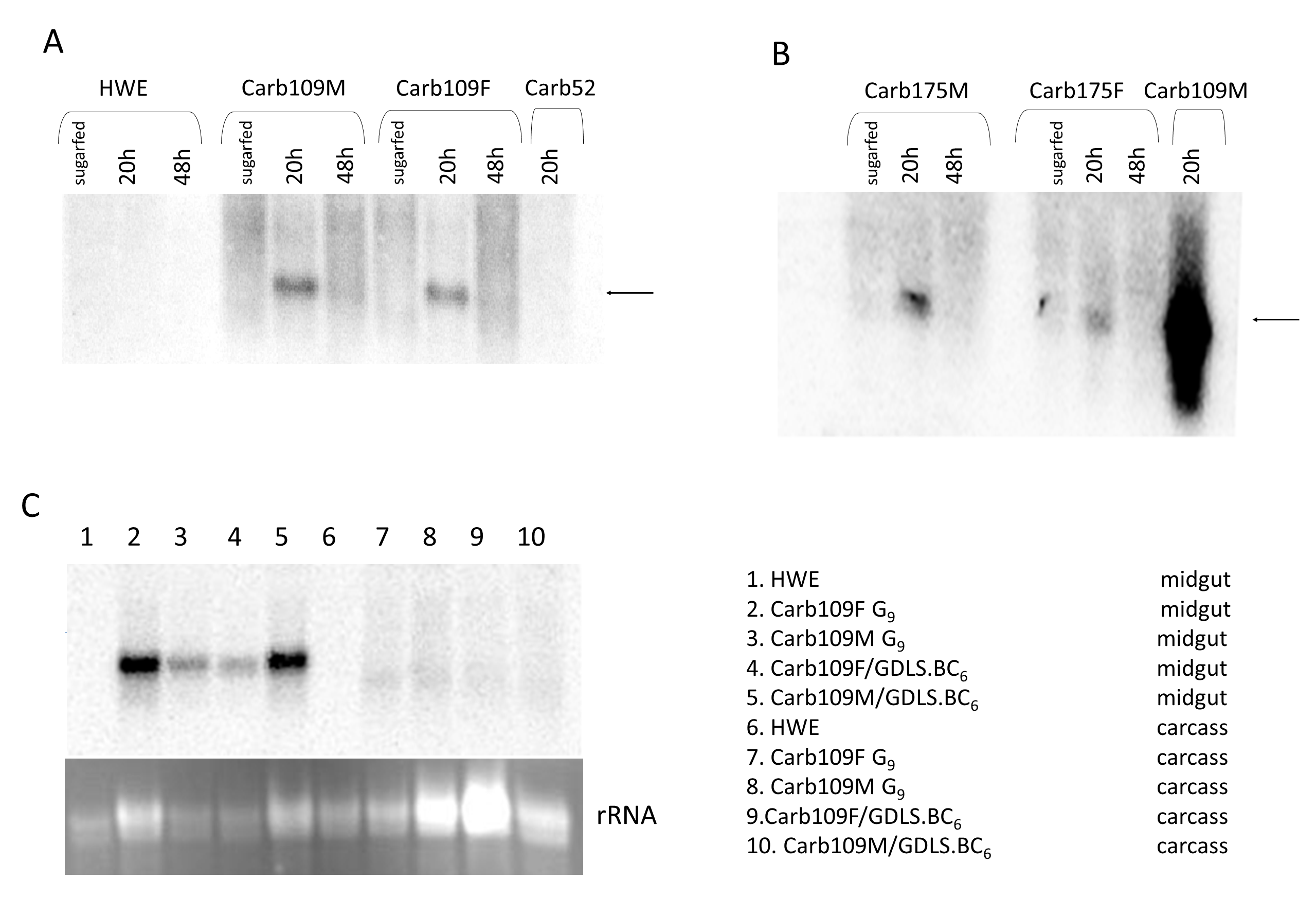

Supplement: Figure S2 — Detection of DENV2-specific IR-RNA isolated from midguts of bloodfed Carb109M, Carb109F, and Carb175F mosquitoes. (A) Detection of IR RNA in midguts of bloodfed females of lines Carb109F and Carb109M. HWE and Carb52 mosquitoes were used as controls. (B) The same effector RNA was detected weakly in midguts of bloodfed Carb175F females. Blots were exposed for 72 h using Carb109M RNA as control. (C) Detection of IR effector RNA at 20 h post-bloodmeal (pbm) in midguts of Carb109F/GDLS.BC6 and Carb109M/GDLS.BC6 (top). The ethidium-bromide stained gel is shown as a loading control (bottom). Blots were hybridized a probe corresponding to the prM-M encoding cDNA of DENV2. Arrow indicates a 500 nt RNA marker. (TIF) [file pntd.0002833.s002.tif]

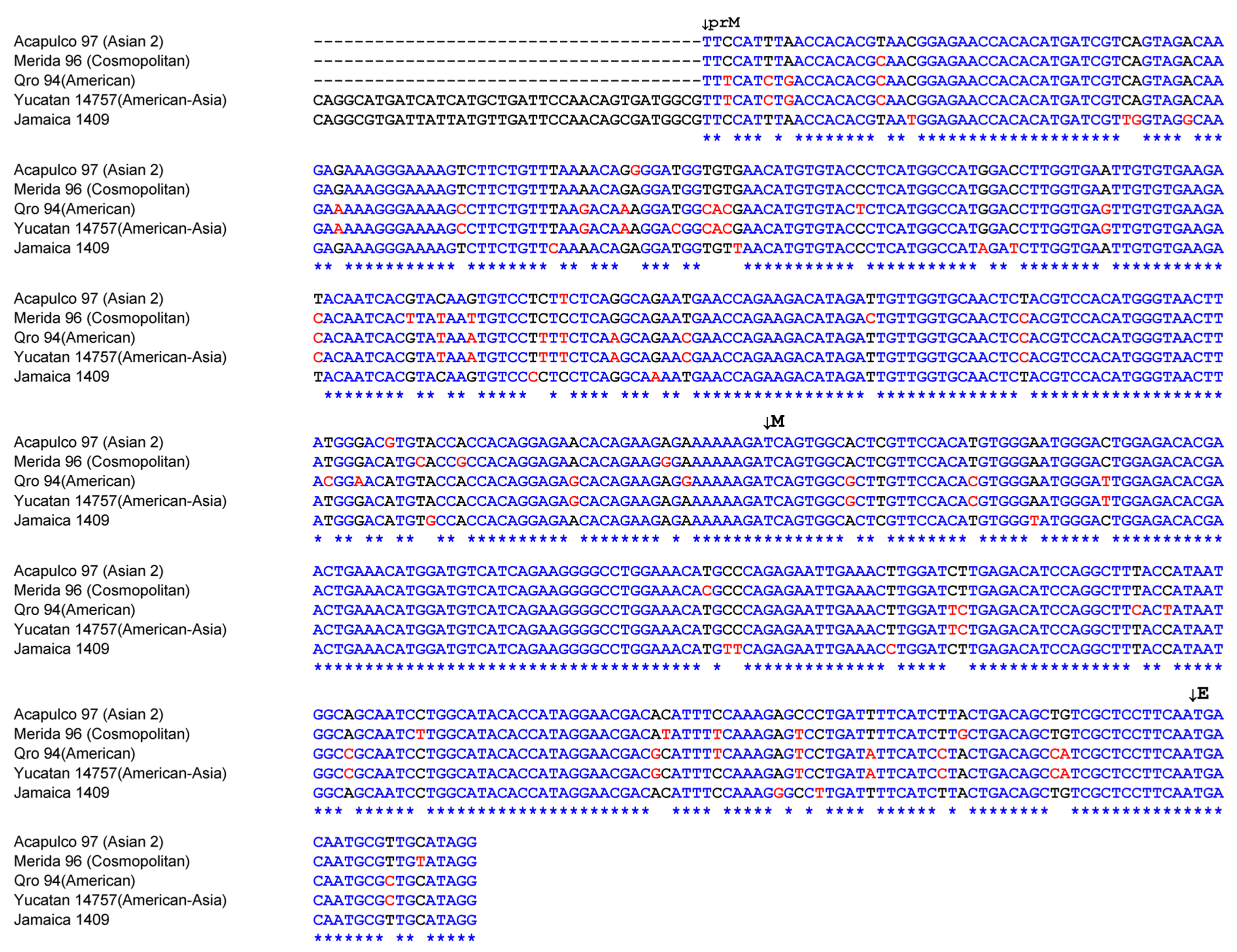

Supplement: Figure S3 — Nucleotide sequence alignment of the 568 nt prM-M encoding region of different DENV2 isolates from Mexico. DENV2 isolates C-932/Acapulco 97, Jam1409, Mex96 Merida, QR94 Quintana Roo, and 14757 Yucatan represent four different DENV2 genotypes targeted by the IR-RNA. RNAi is a homology-dependent antiviral pathway and Carb109M displayed a refractory phenotype to the four viruses. (TIF) [file pntd.0002833.s003.tif]

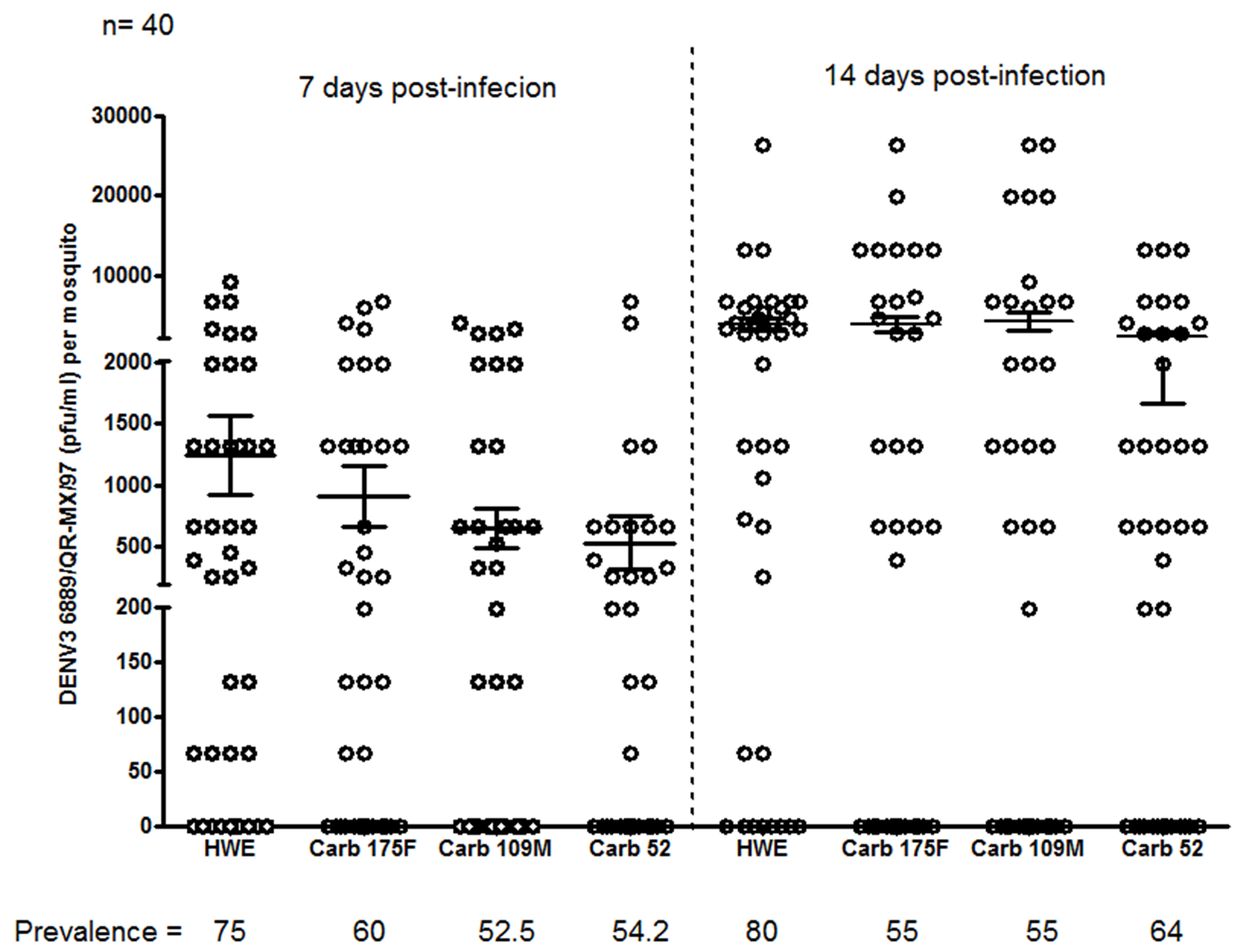

Supplement: Figure S4 — DENV3 challenge of Carb109M mosquitoes. HWE (control), Carb175F, Carb109M, and Carb52 (transgenic control expressing a fluorescent reporter in midgut tissue) mosquitoes received an oral bloodmeal containing 2.3×10^6^ pfu/ml DENV3-6889/QR-MX/97. Virus titers of mosquitoes were assessed at 7 and 14 dpi. Each data point represents the virus titer of a single female. Mean values and standard errors are indicated. (TIF) [file pntd.0002833.s004.tif]

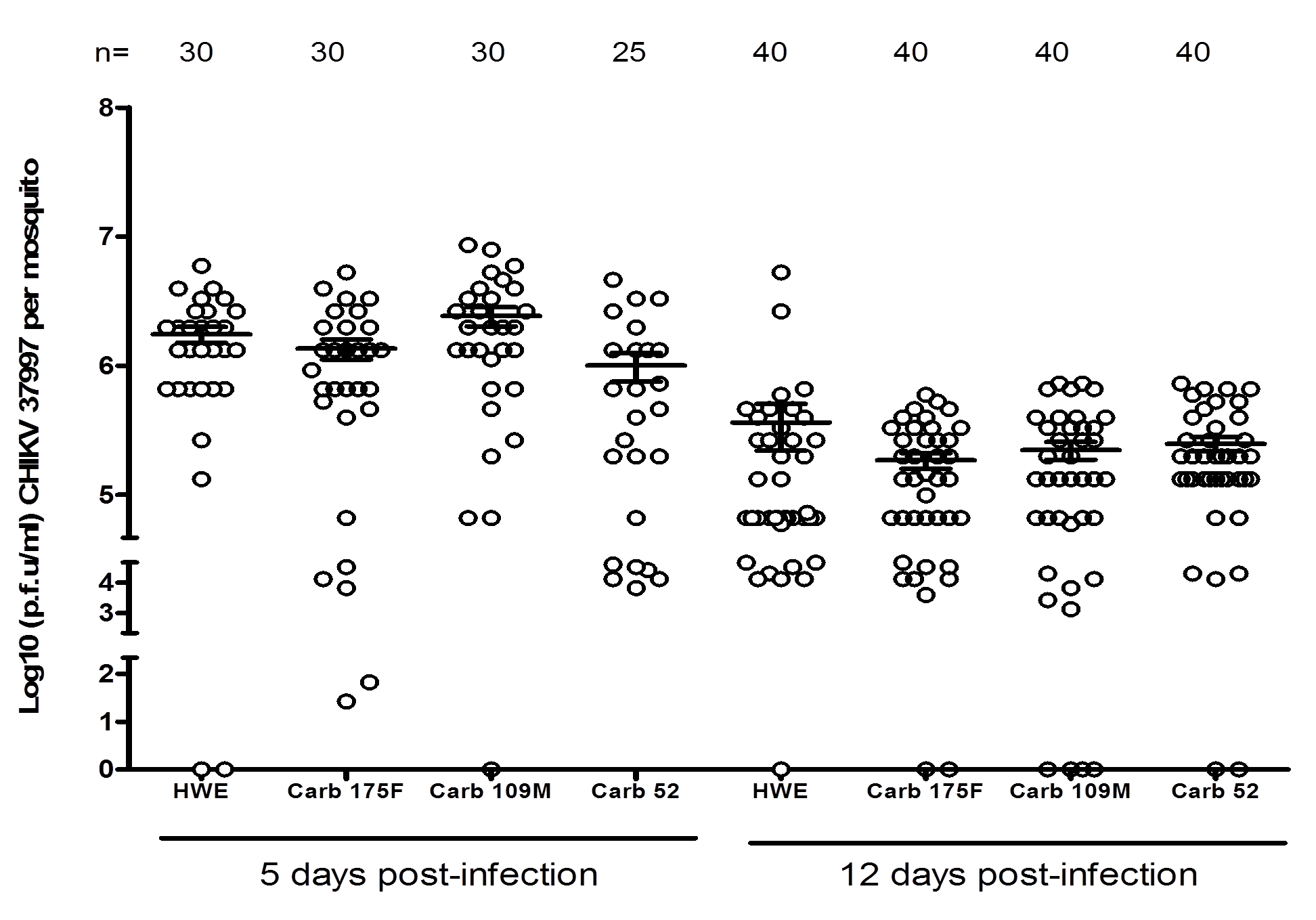

Supplement: Figure S5 — CHIKV challenge of Carb109M mosquitoes. HWE (control), Carb175F, Carb109M, and Carb52 (transgenic control expressing a fluorescent reporter in midgut tissue) mosquitoes received an oral bloodmeal containing 8.13×10^7^ pfu/ml CHIKV 37997. Virus titers of mosquitoes were assessed at 5 and 12 dpi. Each data point represents the virus titer of a single female. Mean values and standard errors are indicated. (TIF) [file pntd.0002833.s005.tif]
